# The temporal trend and distribution characteristics in mortality of Alzheimer's disease and other forms of dementia in China: Based on the National Mortality Surveillance System (NMS) from 2009 to 2015

**DOI:** 10.1371/journal.pone.0210621

**Published:** 2019-01-31

**Authors:** Zhenyan Bo, Yang Wan, Steven Siyao Meng, Tengfei Lin, Weihong Kuang, Lijun Jiang, Peiyuan Qiu

**Affiliations:** 1 West China School of Public Health/No. 4 West China Teaching Hospital, Sichuan University, Chengdu, China; 2 Department of Psychiatry, University of Rochester Medical Center, Rochester, United States of America; 3 Beijing Advanced Innovation Center for Food Nutrition and Human Health, College of Food Science and Nutritional Engineering, China Agricultural University, Beijing, China; 4 West China Hospital, Sichuan University, Chengdu, China; The University of Warwick, UNITED KINGDOM

## Abstract

**Background:**

China is experiencing rapid age, which will lead to increasing burden of age-related diseases, such as Alzheimer disease and other forms of dementia.

**Objectives:**

*T*he aim of this study was to 1) Explore the temporal trend of mortality of Alzheimer disease (AD) and other forms of dementia in China and 2) Analyze its geographic variations and urban-rural differences and calculate the years of life lost (YLLs) from AD and other forms of dementia.

**Data and methods:**

Data were extracted from the National Mortality Surveillance System (NMS). Age-standardized mortalities were calculated with the Western Grade 26 Standard Life List, and the YLLs were calculated using the DALY template provided by the WHO / World Bank global burden of disease (GBD) Working Group. The trends in crude and age-standardized mortality of AD and other forms of dementia were examined using Cochran-Armitage trend test.

**Results:**

In China, the crude mortality from AD and other forms of dementia increased from 2009 to 2015, but the age-standardized mortality decreased. The YLLs of AD and other forms of dementia increased during the study period. The age-standardized mortality in the east was higher than those in the west and middle regions, and the age-standardized mortality in rural areas was higher than that in urban areas.

**Conclusion:**

In China, the age-standardized mortality of AD and other forms of dementia decreased from 2009 to 2015. However, the disease burden from AD and other forms of dementia is becoming heavier due to increasing elderly population. Moreover, there were geographic variations and urban-rural differences in mortality of AD and other forms of dementia in China.

## Introduction

With progressively aging populations around the world, dementia, a clinical syndrome of cognitive impairment or recession highly correlated with age, has severely impacted individuals, families, and societies[1]. The World Alzheimer Report 2016 estimated a global prevalence of people living with dementia of 47 million people, which is projected to almost increase to 74.7 million in 2030 and 131 million in 2050[2]. Dementia is a more serious public health problem in low and middle-income countries (LMICs). According to World Bank classification in 2015, 58% of all people with dementia live in LMIC, a proportion which is projected to rise to 63% in 2030 and 68% in 2050[WHO 2015]. Disease burden of dementia is economically devastating, because people with dementia are often in a state of disability and dependence. World Alzheimer Report 2016 estimated that the cost of supporting dementia worldwide is now US$818 billion and will reach a trillion US dollar by 2018[2]. If we plan to achieve 75% coverage of comprehensive dementia care in high income countries and 50% coverage in low and middle income countries by 2030, the annual costs of dementia will decrease to around 0.5% of total expenditure on public healthcare[3].

In China, due to prolonged life expectancy and growing elderly population, dementia has become an important public health issue. The World Alzheimer Report in 2015 estimated that there were over 9.5 million people with dementia in China, which accounts for 20% of the total number of people in the world with dementia[4]. By 2030, prevalence of people living with dementia in China is expected to rise to over 16 million[2]. Shanghai Aging Study, the first prospective community-based cohort study of cognitive impairment in China, recruited 3,141 participants in 2014, of which 156 were diagnosed with dementia, resulting in a prevalence rate of 5.0% (95% CI 4.3–5.8%). Another population-based prevalence survey in urban and rural communities of four cities in China, including Beijing (northeast), Xi’an (northwest), Shanghai (southeast), and Chengdu (southwest) reported a dementia prevalence rate of 6% [5]. A systematic review published in Lancet showed that the prevalence of dementia increased from 1.8% (95% CI 0·0–44·4) to 2.6% (0.0–28.2) at 65–69 years, and 42.1% (0.0–88.9) to 60.5% (39.7–81.3) at age 95–99 years[6]. Even though many studies found increasing trends in the prevalence of dementia, some researchers challenged the validity of these findings. Wu pointed out that this increasing trend of dementia could be a function of changes in study designs and methodological factors, and the actual prevalence has not significantly increased over the last 30 years. Moreover, based on data from the Chinese Longitudinal Healthy Longevity Survey (CLHLS), a recently published study reported a decreasing incidence of age-standardized CI from 58.77‰ to 10.09‰ (*P*<0.001) from 1998 to 2014 after adjusting for covariates[7]. Unfortunately, it is unclear whether the trend of dementia prevalence is increasing or decreasing.

Although there had been abundant literature about dementia prevalence and incidence trends in the rest of the world, few studies explore temporal trends and geographical variations in disease burden of dementia in China. Peng Yin et al investigated temporal trends and geographical variations of dementia mortality among people aged 65 years and older from 2006 to 2012, using data from the national representative Disease Surveillance Points (DSPs) allocated to 7 geographic regions: east, north, central, south, southwest, northwest, and northeast. They found that compared with northern regions, eastern China had significantly higher mortality rates of dementia (rate ratio 2.28; 95% confidence intervals, 1.45–3.60). The study also found dementia mortality rates decreased by 15% in urban areas but increased by 24% in rural areas over the time period[8].

Peng Yin et al, however, did not fully explore disease burden from dementia. This current study aims to investigate the mortality of Alzheimer's disease and other forms of dementia and its more recent temporal trends, geographical variations, and urban-rural differences from 2009 to 2014. Moreover, this study aims to calculate the years of life lost (YLL) as well, in order to provide an estimation of the disease burden from Alzheimer's disease and other forms of dementia in China.

## Methods

### Source of data

**Fig 1:**
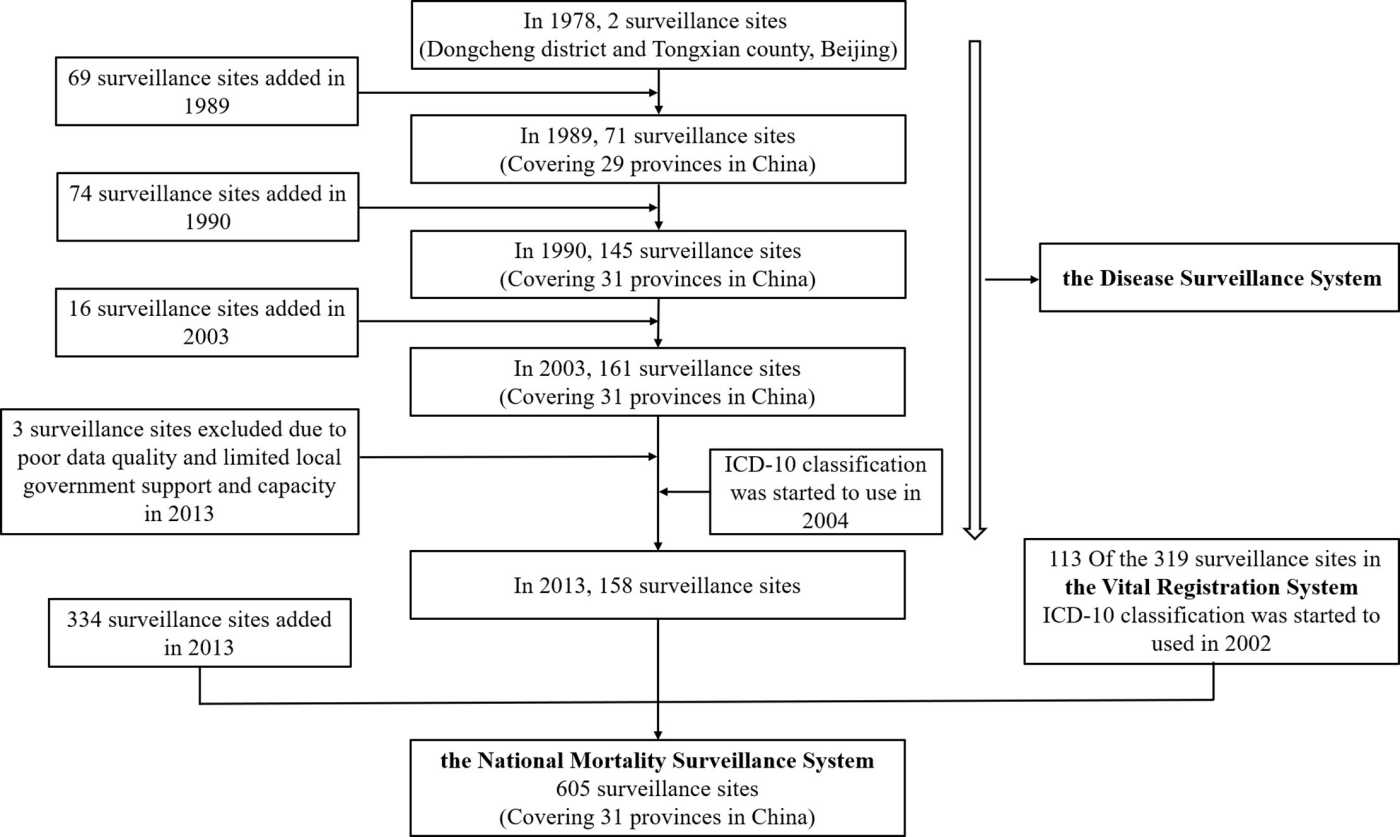
Changes the National Mortality Surveillance System, China.

The data used in this study was gathered from the national representative sample-based Mortality Surveillance System, previously the Disease Surveillance Points system (DSPs). DSPs was primarily proposed by the Peking Union Medical University and Chinese Academy of Medical Sciences in 1978 to collect long-term data on births, causes of death, and the incidence of infectious diseases among the Chinese population. In the same year, a pilot study was carried out at two surveillance sites (East Town and Tongxian) in Beijing[9]. By 1989, driven by the Ministry of Health and guided by the Chinese Academy of Preventive Medicine, the number of surveillance sites had increased to 71 DSPs covering 29 provinces in China. In 1990, an additional 74 surveillance sites were created, this brought the total number of surveillance sites to 145 and included data from approximately 10 million people. In 2003, the system was again expanded to 161 sites, including 64 urban surveillance sites and 97 rural surveillance sites in 31 provinces, autonomous regions and municipalities. Annual monitoring population was more than 77 million, accounting for about 6% of the whole population in China. In 2013, under the guidance of the National Health and Family Planning Commission, 113 of the 319 surveillance sites in the Vital Registration System were selected after meeting strict standards and were added to the DSPs. Meanwhile, another 334 newly selected surveillance sites were added. This brought the total number of surveillance sites to 605, including 207 urban surveillance sites and 398 rural surveillance sites, covering more than 300 million people (about 24% of the Chinese population). In 2013, the DSPs was renamed as the National Mortality Surveillance System. Flowchart of changes in DSPs surveillance sites is shown in Fig 1. All the surveillance sites perform mortality registration work in accordance with the unified standards and procedures described in the guidelines for surveillance in the DSPs[10]. Therefore, the National Surveillance System can provide nationally representative [11] and provincially representative [12] mortality data in China.

Before 2009, central nervous system diseases were classified as Alzheimer's disease, Parkinson and epilepsy in the National Mortality Surveillance System. Nevertheless, from 2009 onwards, mental disorder and central nervous system diseases were combined, AD was recategorized as a type of dementia. Therefore, AD and other form of dementia uses same identification code of U087. In order to keep data consistency, we chose to analyze the mortality data of the AD and other forms of dementia from 2009 to 2015. All diseases were coded in compliance with the rules of the International Classification of Diseases 10 (ICD-10)[13].

### Sampling principles of the National Mortality Surveillance System

The principles of selecting surveillance point of the National Mortality Surveillance System included: 1) Assuring well-balance geographic distribution of surveillance sites; 2) Assuring well-balance distribution of surveillance sites in the following 9 aspects: per-capita gross domestic product (GDP), literacy rate, birth rate, infant death rate, crude death rate, ratio of youth (0–14 years) to total population, ratio of elderly (65 years) to total population, ratio of labor in industry to total population, and ratio of labor in agriculture to total population. Based on the principles above, a multistage stratified random cluster sampling method was used to select surveillance sites[14]. Details about the sampling strategy and quality control measures have been described elsewhere, along with the characteristics of this system and the procedures of collecting data, coding the cause of death and determining the underlying cause of death[9, 14, 15].

### Statistical analysis

All statistical analyses were conducted in IBM spss20.0, and all graphs were drawn in R3.3.0 software. The demographic characteristics used in monitoring the population were described, including gender, area distribution (rural and urban) and region distribution (east, central and west). Age-standardized mortality was calculated using the sixth national census of China as reference population. *χ*^*2*^ test was used to compare the age-standardized mortality between male and female, between rural and urban, and between east, central and west China. Finally, Cochran-Armitage trend test was conducted to explore the temporal trend of mortality of AD and other forms of dementia. YLL was calculated using the DALY template (containing the YLL formula) provided by the WHO / World Bank global burden of disease (GBD) Working Group[16].The standard life expectancy was in accordance with the Western Grade 26 Standard Life List (80.00 for male, 82.50 for female). All statistical tests were 2-tailed, and *P* values of less than 0.05 or 0.013 (when mortality was compared between two different regions) were considered to indicate statistical significance.

## Results

### 1. Basic information

#### 1.1 Distribution of monitoring population

The average annual monitoring population in the National Mortality Surveillance System was about 77 million (before 2013 and more than 300 million after the expansion of the system in 2013). Accordingly, the surveillance population ratio of urban to rural and the ratio of east to central to west changed from1:1.61 to 1:2.15, and from 1.46:1.30:1 to 1.54:1.42:1, respectively. The sex ratio of male to female remained the same, which was 1.04: 1.

#### 1.2 Distribution of deaths from AD and other forms of dementia

During 2009 to 2015, there were 45574 deaths from AD and other forms of dementia registered in the National Mortality Surveillance System. There were 20204 male cases and 25370 female cases with the sex ratio of 1:1.26. The number of deaths in rural area (31391 cases) was more than two times as much as that in urban area (14183 cases) with the ratio of urban to rural of 1:2.21. Compared with the middle and western region, the eastern region had the most AD-related deaths (25892 cases), and the ratio of east to central to west was 3.05:1.32:1(Table 1).

**Table 1:** Distribution characteristic of the deaths from Alzheimer's disease and other forms of dementia, China, 2009–2015.

Year	deaths	gender		area		region		
		male	female	urban	rural	east	middle	west
2009	2377	1089	1288	875	1502	1444	523	410
2010	2845	1231	1614	1039	1806	1577	733	535
2011	2890	1304	1586	1022	1868	1475	810	605
2012	3155	1395	1760	1070	2085	1654	917	584
2013	10654	4690	5964	3103	7551	6072	2789	1793
2014	11466	5119	6347	3421	8045	6652	2773	2041
2015	12187	5376	6811	3653	8534	7018	2651	2518
total	45574	20204	25370	14183	31391	25892	11196	8486

### 2. Crude mortality and age-standardized mortality distribution

After adjusting age, male had a higher mortality of AD and other forms of dementia than their female counterparts (167.085 per 100,000 vs 145.174 per 100,000). The age-standardized mortality difference between males and females was statistically significant (*P*<0.05) in every year, except for 2009 and 2010 (Table 2). http://www.ageing.oxfordjournals.org/Furthermore, rural areas had a significantly higher mortality rate than in urban areas (*P*<0.001) in every year (Table 3). The disparity in total mortality rates between the three geographical regions was also statistically significant (Table 4), and the age-standardized mortality was significantly higher in eastern China (*P*<0.001) (Table 4).

**Table 2:** Crude and age-standardized mortality (1/100,000) of Alzheimer 's disease and other forms of dementia in different genders, China, 2009–2015.

Year	male		female		*χ*^*2*^	*P*
	mortality	age-standardized	mortality	age-standardized		
2009	2.84	4.47	3.51	4.19	3.51	0.061
2010	3.06	5.23	4.18	5.30	0.17	0.680
2011	3.31	5.34	4.17	4.65	18.11	<0.001
2012	3.55	4.00	4.65	3.71	4.30	0.038
2013	4.05	4.12	5.35	3.95	4.07	0.044
2014	3.96	3.83	5.11	3.59	9.53	0.002
2015	4.11	3.97	5.37	3.80	4.54	0.033
total	3.79	4.08	4.94	3.87	29.50	<0.001
*χ*^*2*^	—	167.085	—	145.174	—	—
*P*	—	<0.001	—	<0.001	—	—

**Table 3:** Crude and age-standardized mortality (1/100,000) of Alzheimer 's disease and other forms of dementia in different areas, China, 2009–2015.

Year	urban		rural		*χ*^*2*^	*P*
	mortality	age-standardized	mortality	age-standardized		
2009	3.28	3.57	3.11	5.02	80.866	<0.001
2010	3.48	4.40	3.69	6.00	88.629	<0.001
2011	3.31	4.49	4.01	5.23	20.777	<0.001
2012	3.48	3.25	4.49	4.26	49.502	<0.001
2013	4.41	3.48	4.81	4.34	86.651	<0.001
2014	4.23	3.46	4.66	3.86	23.583	<0.001
2015	4.40	3.59	4.89	4.06	31.141	<0.001
total	4.03	3.58	4.52	4.20	231.034	<0.001
*χ*^*2*^	—	32.334	—	402.737	—	—
*P*	—	<0.001	—	<0.001	—	—

**Table 4:** Crude and age-standardized mortality (1/100,000) of Alzheimer 's disease and other forms of dementia in different regions, China, 2009–2015.

year	East		Central		West		*χ*^*2*^	*P*
	Mortality	age-standardized	Mortality	age-standardized	Mortality	age-standardized		
2009	5.03	5.85	2.02	3.04	2.01	3.03	338.661	<0.001
2010	5.13	6.40	2.75	4.29	2.51	4.39	156.326	<0.001
2011	4.89	5.46	2.99	4.39	3.00	4.66	35.855	<0.001
2012	5.51	4.49	3.39	3.48	2.91	3.12	70.096	<0.001
2013	6.92	5.07	3.38	3.29	3.14	3.03	492.547	<0.001
2014	6.67	4.74	3.09	2.83	3.18	2.96	578.133	<0.001
2015	7.11	5.05	2.97	2.68	3.62	3.41	727.765	<0.001
tall	6.38	4.99	3.04	3.20	3.11	3.18	2253.902	<0.001
*χ*^*2*^	—	92.578	—	184.628	—	34.374	—	—
*P*	—	<0.001	—	<0.001	—	<0.001	—	—

### 3. Age trend of mortality of AD and other forms of dementia

**Fig 2:**
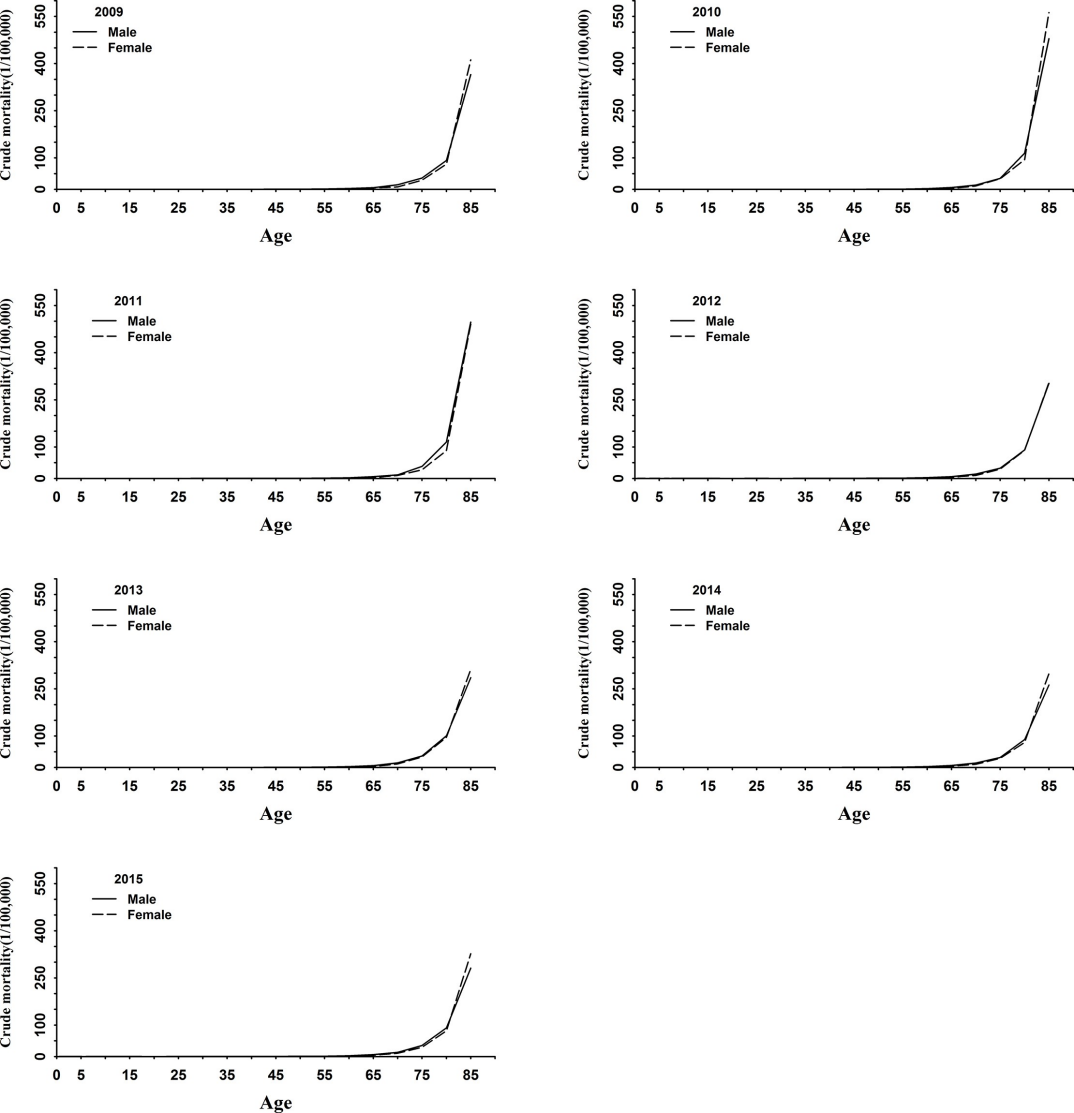
Age trend of Alzheimer's disease and other forms of dementia in China, 2009–2015.

The mortality of Alzheimer's disease and other forms of dementia increased with age in both male and female throughout all years (Fig 2). As shown in the figure, the mortality from AD and other forms of dementia was very low before 65 years, but increased significantly after 65 years of age. After the age of 80, the mortality suddenly increased sharply and reached the apex soon after.

### 4. Mortality trend of AD and other forms of dementia

**Fig 3:**
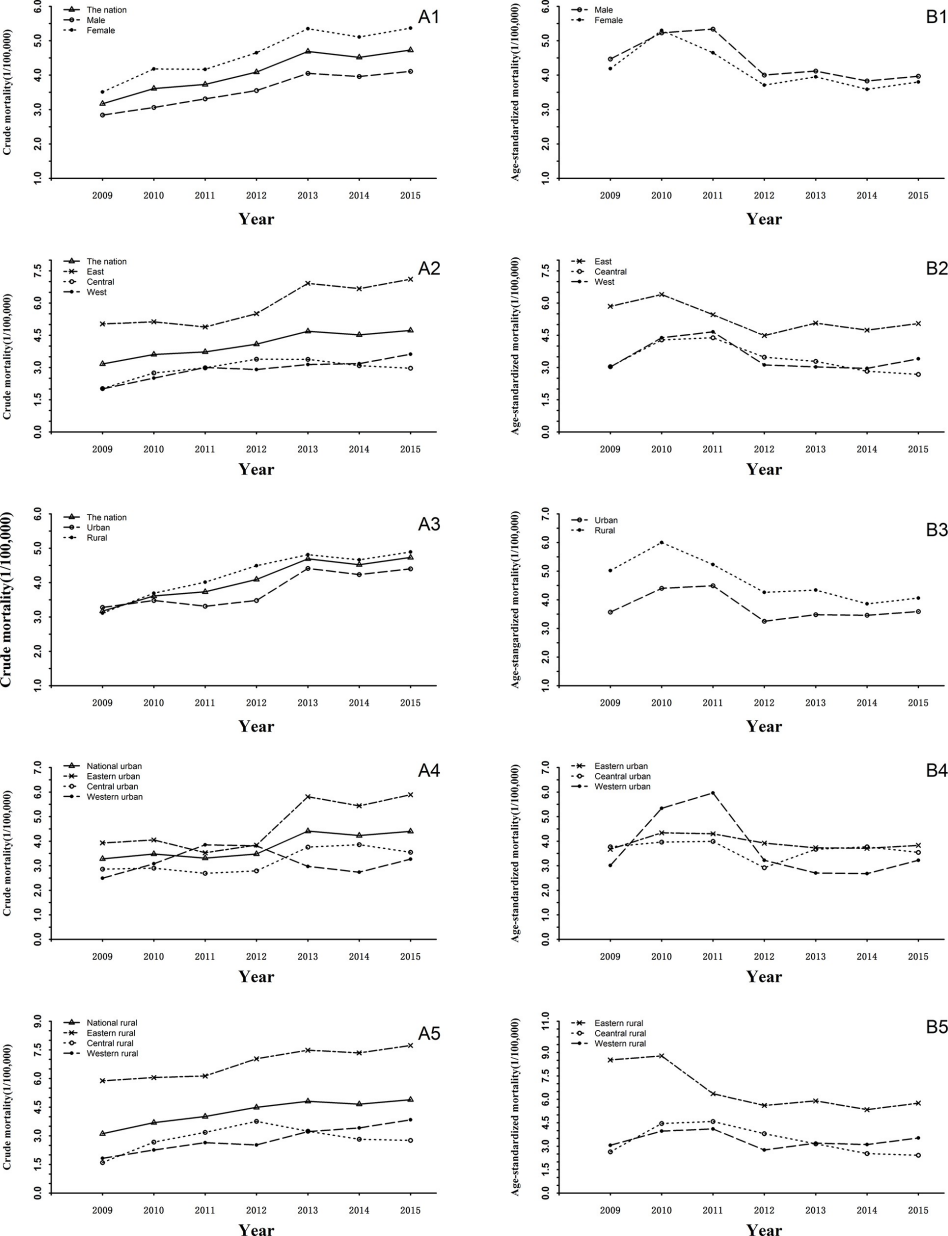
Time trend of mortality of Alzheimer's disease and other forms of dementia from 2009 to 2015, China. A1: crude mortality in male and female. B1: age-standardized mortality in male and female. A2: crude mortality in eastern, middle and western China. B2: age-standardized mortality in eastern, middle and western China. A3: crude mortality in rural and urban China. B3: age-standardized mortality in rural and urban China. A4: crude mortality in urban area of eastern, middle and western China B4: age-standardized mortality in urban area of eastern, middle and western China. A5: crude mortality in rural area of eastern, middle and western China. B5: age-standardized mortality in rural area of eastern, middle and western China.

The crude mortality increased from 2009 to 2015 (Fig 3A1–3A3). However, after standardizing by age, the data showed a different trend. The mortality initially increased from 2009 to 2010. Thereafter, there was a trend of decrease in mortality with slight fluctuations. This declining trend over time was significant (*P*<0.001)for both sexes (Fig 3B1) and three regions (Fig 3B2). Similar pattern of mortality trend of AD and other forms of dementia was observed in urban and rural areas (Fig 3B3).

We further analyzed the temporal trend of mortality in eastern urban, central urban, and western urban areas, as well as mortality in eastern rural, central rural, and western rural areas (Fig 3B4 and 3B5). In the urban setting, decreasing mortality trends over time was significant in the western area (*P*<0.001), but insignificant in eastern and central areas (*P* = 0.117, 0.229). While in rural areas, statistically significant decreasing trends (*P*<0.001) were observed in both eastern and central area (Fig 3B5). For western rural areas, decreasing trend of AD and other forms of dementia was not significant (*P* = 0.220).

### 5. Disease burden (YLLs)

**Fig 4:**
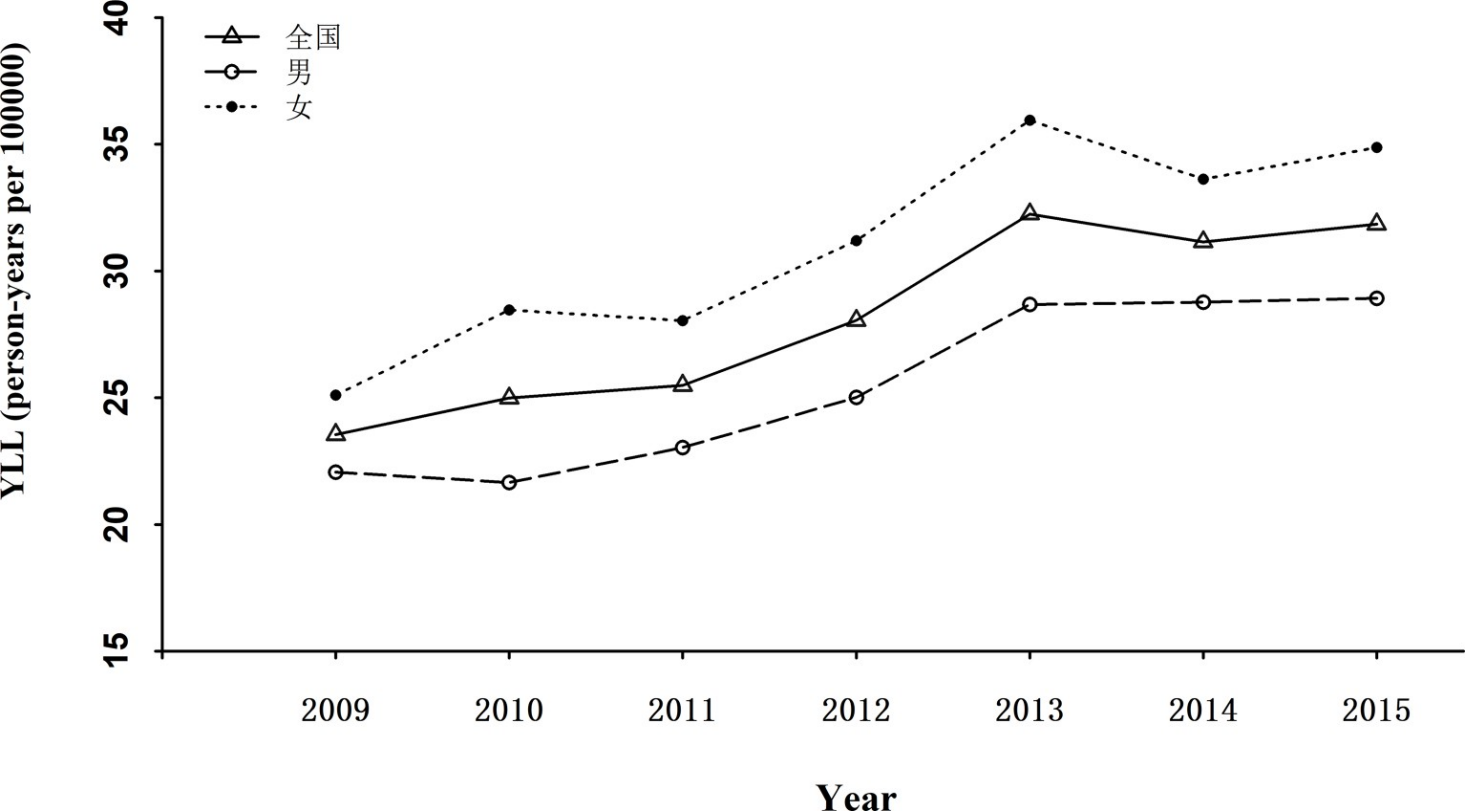
Secular trend of YLL caused by Alzheimer's disease and other forms of dementia in China, 2009–2015.

From 2009 to 2015, the average YLLs caused by AD and other forms of dementia was 29.91 person-years per 100000. The disease burden in female (32.85 person-years per 100000) was higher than that in male (27.07 person-years per 100000). Between different regions, the YLLs varied in a wide range from 21.75 person-years per 100000 in central region to 42.19 person-years per 100000 in eastern region (Table 5). The average YLLs were 27.81 person-years per 100000 and 30.97 person-years per 100000 in urban and rural areas, respectively, suggesting disease burden caused by AD and other forms of dementia was heavier in rural area than in urban area. Regardless of urban or rural settings, within the three regions, disease burden was the heaviest in eastern China (Table 5). Overall, the disease burden caused by AD and other forms of dementia showed an upward trend (Fig 4) during the study period.

**Table 5:** YLLs of Alzheimer 's disease and other forms of dementia (person-year per 100,000), 2009–2015.

		Male	Female	Total
Total		27.07	32.85	29.91
Area				
	Urban	25.40	30.29	27.81
	Rural	27.92	34.16	30.97
Region				
	East	36.17	48.38	42.19
	Central	20.92	22.62	21.75
	West	21.94	23.36	22.63
Urban				
	East	30.24	37.76	33.96
	Central	22.52	26.24	24.36
	West	20.71	22.46	21.57
Rural				
	East	39.75	54.86	47.18
	Central	20.27	21.10	20.67
	West	22.55	23.16	23.16

## Discussion

Using a nationally representative data set, this study found that the age-standardized mortality of AD and other forms of dementia showed a decreasing trend of prevalence from 2010 to 2015. Eastern China had the highest mortality when compared with the central and western regions. The mortality in rural areas was higher than in urban areas; the mortality was higher in men than in women.

In Europe and America, the prevalence of dementia was reported to be stable or decreasing over the last decades, attributing to secular improvement of educational level, population-level reduction in vascular risk factors, and overall reduction in stroke incidence[17–21].However, a number of studies showed an increasing trend in dementia prevalence in Mainland China and Hong Kong[22–24]. Meanwhile, there are some research reporting stable trends in prevalence of dementia or decreasing trends in the incidence of cognitive impairment[7]. A multicenter study[25] showed a stable trend in prevalence of AD and other dementias in China. This study also attributed the observed increasing trend in other studies to the inconsistent study designs and methodological factors such as dramatic changes in diagnostic criteria in the past 30 years[26]. In addition, there have been some studies in recent years reporting that the actual trend of dementia mortality is decreasing. One prior study using DSPs data from 2006 to 2012 found the age adjusted mortality of dementia decreased from 39.6 to 33.7 in urban areas of China[8]. This study is consistent with our findings that the age-standardized mortality of AD and other forms of dementia decreased from 2009 to 2015 in China[27]. The decline in mortality may be attributed to better efforts for active prevention and treatment of dementia-related diseases. Recent data from the Rotterdam Study also confirmed a strong relationship between declining incidence of dementia and preventive measures to better control and treatment of vascular risk factors[28]. But due to the current limitation of the data, we cannot determine conclusive reasons for the downward trend of age-adjusted mortality of AD and other forms of dementia in China.

In our study, we found a higher mortality of AD and other forms of dementia in rural areas than in urban areas of China, which is consistent with previous studies[25, 29, 30]. This gap between rural and urban areas may be attributed to the differences in education level. Prior evidence supports that education level plays an important role in the development of AD and other forms of dementia. People with better education are less likely to suffer from AD and other forms of dementia [6, 25]. In China, people in rural areas have less opportunity to receive education than their counterparts living in urban areas, especially those born in 1950s who are now in their 60s. Jianping Jia and his colleagues[25] found that, among the older population in China, aged 65 and above, the proportion of illiterate individuals in rural areas was 48.2%, which is much higher than in urban areas (17.7%).

Consistent with previous studies[8], we found a far higher mortality rate in eastern regions of China. Since adaptation of reform and opening policy, the process of urbanization in the eastern region was accelerated at an unimaginable speed, transforming it into the most economically prosperous region in China. However, due to the changing environment and fast pace of life, people's lifestyle in the region had undergone tremendous changes, including development of poorer sleep hygiene and unhealthy eating habits. These changes increased the risk of suffering from various chronic diseases, like hypertension, diabetes and stroke, which are highly correlated with onset of Alzheimer's disease and other dementia[31–33]. A recent study revealed that residents of eastern China were more susceptible to Alzheimer's disease and other forms of dementia, due to associated chronic disease risk factors [34]. The national chronic disease surveillance report in 2010 also showed that the prevalence of hypertension, overweight and obesity, diabetes, and hyperlipidemia in eastern China was the highest.

As reported by the World Alzheimer Report in 2010, the societal costs of AD and other dementia is almost as high as those of cancer, heart disease and stroke[35]. Due to the increasing global aging population, societal costs will likely skyrocket in the future. In this study, we computed YLLs to measure the premature mortality burden caused by AD and other forms of dementia in China. In India, the overall YLL in 2007–2008 was 47.13 person-years per 100000[36], which is higher than the YLL in our study (29 .91 person-years per 100000). It has been estimated that China has the same population of AD patients as both Europe and North America combined, indicating heavy disease burden caused by AD in China [37, 38]. In this study, we found that the age-adjusted mortality of AD and other forms of dementia was decreasing, but the disease burden was on the rise. The results indicated that disease burden of AD and other forms of dementia is increasing due to increasing number of people living with AD and other forms of dementia, and the primary reason is population aging. Considering the rapid aging in China, we think the disease burden caused by AD and other forms dementia will be increasing rapidly in the coming decades [39].

Our current study had certain limitations that might have affected the accuracy of our results. Though the data used in the study was nationally presentative and the quality of the data is guaranteed by a number of regulatory measures, we cannot obtain the individual level information that might have impacted the mortality of AD and other forms of dementia. Therefore, we cannot carry out further analysis to explore the explanatory factors, such as education level, health status, and marital status in relation to spatiotemporal variations. Second, the National Mortality Surveillance System counts cases based on the most immediate cause of death. If a person suffering AD and other forms of dementia died of other acute causes, such as pneumonia and fall trauma, then the system will code the cause of death under these acute conditions rather than AD and other forms of dementia. This might underestimate the true mortality rates for AD and other forms of dementia. Lastly, in this study, without incidence data of AD and other forms of dementia, we are unable to calculate the total DALYs (disability adjusted life years) caused by AD and other forms of dementia, which is a more comprehensive and accurate measurement of the disease burden.

In conclusion, this study has demonstrated the spatiotemporal variation of mortality of AD and other forms of dementia from 2009 to 2014 in China. The crude mortality of AD and other forms of dementia increased during the study period. In addition, the burden of disease caused by AD and other forms of dementia also increased. However, after adjusting for age, there was a decreasing trend in mortality for AD and other forms of dementia, suggesting that the increasing aging population could explain the observed trend of in crude mortality seen before. The findings of this study also reflect urban-rural and regional differences. The age-standardized mortality in the east was higher than that in the west and middle regions, and age-standardized mortality in rural areas was higher than in urban areas. The government and relevant departments should carry out reasonable and effective disease prevention and control measures based on the distribution characteristics of AD and other forms of dementia to provide better treatment and caring service for the patients.
